# The Inflammatory Cytokine Profile of Patients with Malignant Pleural Effusion Treated with Pleurodesis

**DOI:** 10.3390/jcm9124010

**Published:** 2020-12-11

**Authors:** Li-Han Hsu, Thomas C. Soong, Nei-Min Chu, Chung-Yu Huang, Shu-Huei Kao, Yung-Feng Lin

**Affiliations:** 1 Ph.D. Program in Medical Biotechnology, College of Medical Science and Technology, Taipei Medical University, Taipei 11031, Taiwan; lhhsu@kfsyscc.org (L.-H.H.); d609105006@tmu.edu.tw (C.-Y.H.); 2 Division of Pulmonary and Critical Care Medicine, Sun Yat-Sen Cancer Center, Taipei 11259, Taiwan; 3 Department of Medicine, School of Medicine, National Yang-Ming University, Taipei 11221, Taiwan; 4 Department of Radiology, Sun Yat-Sen Cancer Center, Taipei 11259, Taiwan; clsoong@kfsyscc.org; 5 Department of Medical Oncology, Sun Yat-Sen Cancer Center, Taipei 11259, Taiwan; nmchu@kfsyscc.org; 6 School of Medical Laboratory Science and Biotechnology, College of Medical Science and Technology, Taipei Medical University, Taipei 11031, Taiwan

**Keywords:** cytokine, inflammation, malignant pleural effusion, pleurodesis, survival

## Abstract

Patients with malignant pleural effusion (MPE) who underwent successful pleurodesis survive longer than those for whom it fails. We hypothesize that the therapy-induced inflammatory responses inhibit the cancer progression, and thereby lead to a longer survival. Thirty-three consecutive patients with MPE that were eligible for bleomycin pleurodesis between September 2015 and December 2017 were recruited prospectively. Nineteen patients (57.6%) achieved fully or partially successful pleurodesis, while 14 patients either failed or survived less than 30 days after pleurodesis. Two patients without successful pleurodesis were excluded because of missing data. Interleukin (IL)-1 beta, IL-6, IL-10, transforming growth factor beta, tumor necrosis factor alpha (TNF-α), and vascular endothelial growth factor in the pleural fluid were measured before, and after 3 and 24 h of pleurodesis. Their pleurodesis outcome and survival were monitored and analyzed. Patients who underwent successful pleurodesis had a longer survival rate. Patients without successful pleurodesis had significantly higher TNF-α and IL-10 levels in their pleural fluid than in the successful patients before pleurodesis. Following pleurodesis, there was a significant increment of IL-10 in the first three hours in the successful patients. In contrast, significant increments of TNF-α and IL-10 were found in the unsuccessful patients between 3 and 24 h after pleurodesis. The ability to produce specific cytokines in the pleural space following pleurodesis may be decisive for the patient’s outcome and survival. Serial measurement of cytokines can help allocate the patients to adequate treatment strategies. Further study of the underlying mechanism may shed light on cytokine therapies as novel approaches.

## 1. Introduction

Pleurodesis is regarded as a symptomatic treatment to prevent fluid re-accumulation in patients with malignant pleural effusion (MPE) [1,2]. Talc pleurodesis had been demonstrated as having a favorable impact on the survival of patients with MPE [3,4]. Tremblay et al. showed that patients who spontaneously pleurodesed after indwelling pleural catheter placement survived longer [5]. Ren et al. reported that the intrapleural staphylococcal superantigen has a survival benefit in addition to the resolution of MPE in patients with non-small cell lung cancer [6]. Recently, we showed that patients who underwent successful minocycline pleurodesis had a longer cancer-specific survival than those for whom it failed [7,8]. The successfully induced inflammatory response is proposed to prohibit the tumor invasion and metastasis, rather than simply through the physical barrier by the fibrin formation [8].

There are continued improvements to our understanding of the molecular connections between the inflammation and cancer [9,10]. While chronic inflammation might promote tumor formation, acute inflammation may well hamper the process, and is indeed used therapeutically to inhibit the tumor [10,11]. Cytokines are signaling molecules that are key mediators of inflammation. They can be generally classified as pro-inflammatory or anti-inflammatory, and as tumorigenic or tumor suppressive [12]. Interleukin-1 beta (IL-1β), IL-6, IL-10, transforming growth factor beta (TGF-β), and tumor necrosis factor alpha (TNF-α) are representative cytokines that are important modulators of inflammation and cancer progression [9,10]. Interleukin-6 and TNF-α are usually reported as pro-inflammatory cytokines. Interleukin-10 is a cytokine with anti-inflammatory properties. Interleukin-1 beta and TGF-β have dual function and pleiotropic nature. Vascular endothelial growth factor (VEGF) is a potent stimulator of angiogenesis and the mediator of the pleural fluid formation [13].

In the study, serial measurements of IL-1β, IL-6, IL-10, TGF-β, TNF-α, and VEGF in pleural fluid before and after chemical pleurodesis were performed prospectively in patients with MPE. They were correlated with the pleurodesis outcome and survival. We tried to find the differences in the cytokine profile between patients that succeeded in pleurodesis and those that did not succeed, and identify the cytokine decisive for the prognosis.

## 2. Material and Methods

### 2.1. Patients and Pleurodesis

Consecutive patients with symptomatic MPE that were eligible for chemical pleurodesis at the Sun-Yat Sen Cancer Center, a 200-bed hospital, were prospectively recruited between September 2015 and December 2017. To reduce the confounding factors, patients underwent intrapleural urokinase therapy for loculated MPE or a trapped lung before pleurodesis was excluded [8,14]. Loculated MPE was defined as fluid collections with septa seen on chest computed tomography and/or ultrasonography or air–fluid levels in the pleural space on the chest radiograph. A trapped lung was suggested by mechanical restriction of the visceral pleura preventing lung expansion. All the patients received treatment for the underlying primary tumors according to the current guidelines and were followed by the medical oncologists. All the recruited subjects signed an informed consent for the procedures and the laboratory study. The institutional review board of the Sun Yat-Sen Cancer Center approved this study (No. 20160223A and No. 20170220A). The study was also approved by the ethics committee of the Sun Yat-Sen Cancer Center, and it was conducted in accordance with the ethical principles stated in the Declaration of Helsinki or the guidelines on good clinical practice.

Since the discontinuation of the commercial production of minocycline in Taiwan during the study period, bleomycin has been adopted as the sclerosing agent [15]. In contrast to the talc poudrage, which cannot be blown through a catheter, and slurry accumulation in the dependent areas, bleomycin allows for a more even distribution in the pleural space. A size eight to 14 Fr self-retaining intrapleural catheter (SKATERTM Single step drainage set; Argon Medical Devices, Athens, TX, USA) was inserted. Pleurodesis was indicated on near-complete ipsilateral lung re-expansion when the daily drainage decreased to less than 150 mL for two consecutive days. Eligible patients were infused with 60 IU bleomycin (Nippon Kayaku Co. Ltd., Tokyo, Japan) diluted in 100mL sterile saline via the intrapleural catheter. The catheter was clamped for three hours after the instillation of bleomycin, and then reopened for suction. Patients were encouraged to change positions during the treatment to facilitate the mixing of the bleomycin with the pleural fluid.

The LENT prognostic score, concerning the lactate dehydrogenase level in the pleural fluid, the Eastern Cooperative Oncology Group performance score, serum neutrophil-to-lymphocyte ratio, and the tumor type were evaluated for each patient [16]. The driver oncogene status of the lung adenocarcinoma, such as the epidermal growth factor receptor (EGFR) mutations, the echinoderm microtubule-associated protein like 4-anaplastic lymphoma kinase (EML4-ALK) translocation, and the estrogen receptor/progesterone receptor/human EGFR 2 status of breast cancer were recorded.

### 2.2. Pleural Fluid Sample Collection

In concordance with the daily practice, a pleural fluid sample was obtained through the intrapleural catheter immediately before the pleurodesis, three hours at the reopening of the catheter, and 24 h later, prior to the removal of the catheter. The pleural fluid sample (up to 15 mL) was immediately centrifuged at 10,000× *g* for 10 min to remove the cell debris and aggregates, and then stored at −80 °C until measurement.

### 2.3. Measurement of Pleural Fluid Inflammatory Cytokines and VEGF

The levels of IL-1β, IL-6, IL-10, TGF-β, TNF-α and VEGF in the supernatants were measured by Bio-Plex^®^ Pro Human Cytokine Multiplex assays (Bio-Rad Laboratories, Hercules, CA, USA) with MagPlex beads in a flat bottom microtiter plate according to the manufacturer’s instructions. Antibody-coupled capture beads were prepared and plated. The plate was again incubated on a shaker and streptavidin-phycoerythin solution was added to the wells. After a last incubation step, beads were resuspended in assay buffer and the absorbance was measured with a MagPix (Luminex Corporation) using the xPONENT software (Luminex Corporation, Austin, TX, USA).

### 2.4. Assessment of the Pleurodesis Outcomes and Analysis of the Survival

Follow-up chest radiographs were obtained at one, three, and six months after pleurodesis and repeated as and when required. The success or failure of pleurodesis was determined according to the relevant definitions proposed by the American Thoracic Society and the European Respiratory Society Consensus Statement [1]. Complete success was defined as the long-term relief of symptoms related to the effusion, with the absence of the fluid re-accumulation on the chest radiograph until death. Partial success was defined as the diminution of the dyspnea related to the effusion, with only partial re-accumulation of fluid (less than 50% of the initial level), with no further therapeutic thoracenteses required for the remainder of the patient’s life. Lack of success, as previously mentioned, was determined as failed pleurodesis.

Fair-to-moderate inter-observer agreements about the definition of the non-expandable lung have been reported [17]. Independent interpretation of the pleurodesis outcome by two assessors (T.C.S. and L.-H.H.), followed by the consensus judgement, was completed in the study to reduce the observer bias.

The survival time was calculated from the date of diagnosis of MPE and censored at the date of death or the last follow-up. The overall survival rate was compared between the patients that had succeeded and failed the pleurodesis.

The baseline values and changes in IL-1β, IL-6, IL-10, TGF-β, TNF-α, and VEGF levels in the pleural fluids were compared between the patients with successful pleurodesis and those without. Because the pleural fluid concentrations of IL-1β, IL-6, IL-10, TGF-β, TNF-α, and VEGF were highly variable in patients with MPE [18], we also used the fold change within the individual patients as a comparison [19]. In addition to comparison between groups, comparison was also made at different time points within each group. Linear regression analyses were performed to measure the correlations between different cytokines before and 24 h after pleurodesis, and were presented with Pearson’s correlation coefficient with the significance level.

### 2.5. Statistical Analysis

Descriptive statistics of mean, median, standard deviation, and frequency were used to process the demographic and laboratory data. Continuous variables were compared using the one-way ANOVA on ranks with SigmaPlot 14.0 (Systat Software, Inc.; San Jose, CA, USA), whereas categorical variables were compared using the chi-square test or Fisher’s exact test. A *p* value of less than 0.05 for comparisons was considered to represent statistical significance. Survival estimates were derived by the Kaplan–Meier plots, while log-rank tests were used to assess the differences in survival among the groups using the statistical software package SAS, version 9.4 (SAS Institute; Cary, NC, USA).

## 3. Results

### 3.1. Patients’ Characteristics and Pleurodesis Outcomes

There were 84 patients diagnosed with MPE in the study period. Thirty-three patients underwent simple bleomycin pleurodesis, and 19 patients (57.6%) achieved successful or partially successful pleurodesis (10 with breast cancer and nine with lung adenocarcinoma). Nine patients failed the pleurodesis (three with lung adenocarcinoma, three with breast cancer, one with small cell lung cancer, one with ovarian cancer, and one with bladder urothelial carcinoma), with pleural fluid re-accumulation before death. Five patients survived less than 30 days after pleurodesis, with a shorter follow-up to evaluate the pleurodesis outcome (two with breast cancer, one with small cell lung cancer, one with ovarian cancer, and one with gastric cancer). The age, gender, and smoking history appeared comparable among the patients who succeeded or failed pleurodesis or survived less than 30 days (Table 1). Patients who underwent successful pleurodesis, failed the pleurodesis, and survived less than 30 days had a mean value of LENT score, 2.84, 3.67, and 4.40, respectively (*p* = 0.020).

### 3.2. Survival Differences

**Figure 1:**
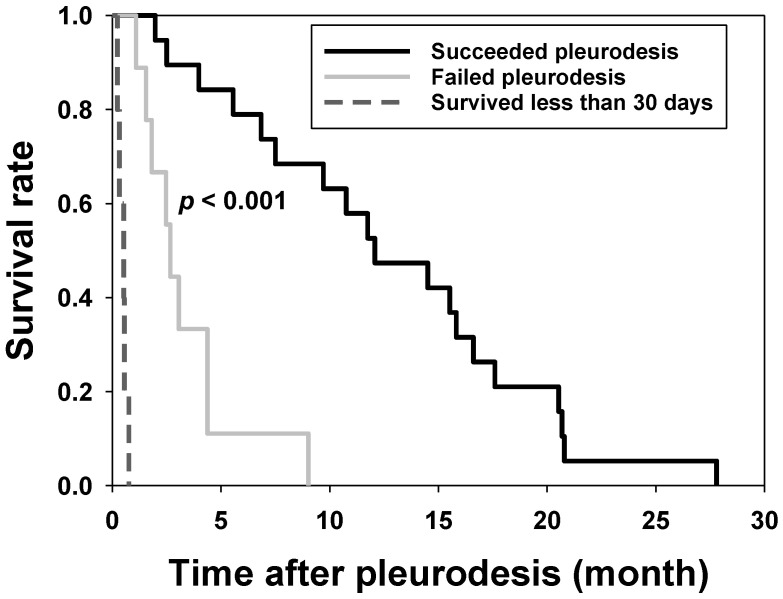
Survival after pleurodesis. The survival rates of patients who underwent successful pleurodesis (black line), patients for whom pleurodesis failed (grey line), and patients who survived less than 30 days (dotted line) were plotted against the time after pleurodesis.

On the follow-up, patients who underwent successful pleurodesis had a significantly longer overall survival than those for whom it failed (median, 367 vs. 81 days; *p* < 0.001) (Figure 1).

### 3.3. Pleural Fluid Inflammatory Cytokines and VEGF between Groups

Two patients with breast cancer had dry drainage at the 24 h collection that led to missing data at the time point and were excluded from the subsequent analysis—one had failed pleurodesis and the other survived less than 30 days. We combined the remaining patients with failed pleurodesis and the patients who survived less than 30 days as a group (*n* = 12) in the subsequent cytokine analysis, so as to compare them with the successful pleurodesis group (*n* = 19), considering the obvious prognostic difference and aim to investigate the survival benefit of the patients who succeeded pleurodesis.

**Figure 2:**
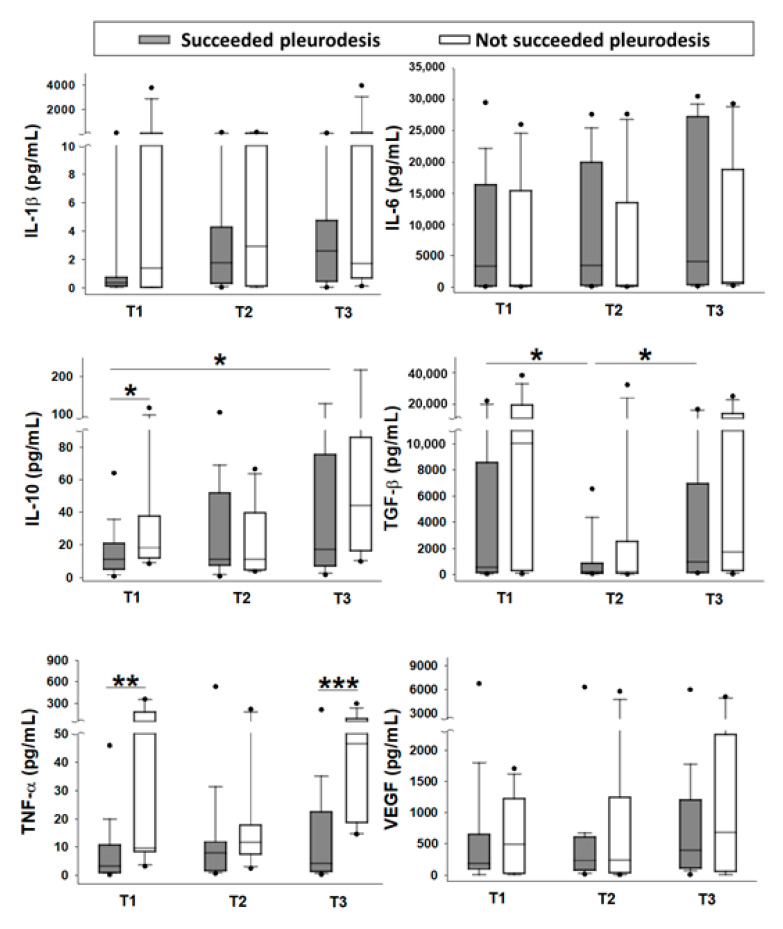
Measured cytokines in the pleural fluid at different time points. Interleukin (IL)-1β, IL-6, IL-10, transforming growth factor beta (TGF-β), tumor necrosis factor alpha (TNF-α), and vascular endothelial growth factor (VEGF) levels were compared between patients who succeeded pleurodesis (grey column; *n* = 19) and those not succeeded (white column; *n* = 12). Comparison was also made at different time points within each group. Box plots demonstrate the concentrations in the pleural fluid. The dots represent the 5th and 95th percentiles, respectively. The error bars cover the 10th to 90th percentiles and the box covers 25th to 75th percentiles. The line within the box represents the median value. T1, before pleurodesis; T2, three hours after pleurodesis; T3, 24 h after pleurodesis; * *p* < 0.05; ** *p* < 0.01; *** *p* < 0.001.

**Figure 3:**
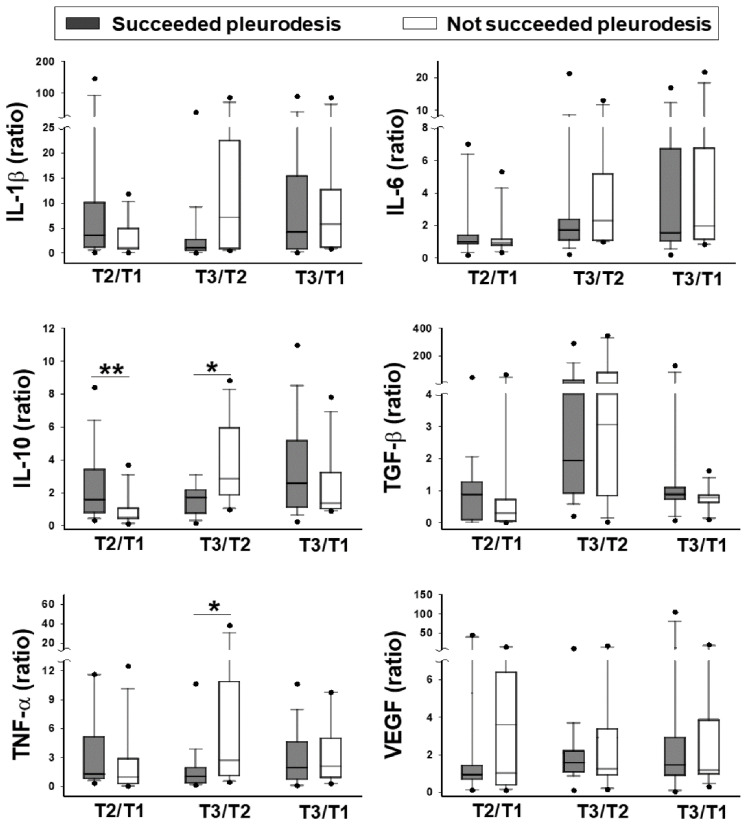
Fold change of the cytokines in the pleural fluid. IL-1β, IL-6, IL-10, TGF-β, TNF-α, and VEGF levels measured at the later time points were divided by those measured at the earlier time points and compared between patients who underwent successful pleurodesis (grey column; *n* = 19) and those not succeeded (white column; *n* = 12). Box plots demonstrated the ratios. The dots represent the 5th and 95th percentiles, respectively. The error bars cover the 10th to 90th percentiles and the box covers the 25th to 75th percentiles. The line within the box represents the median value. T1, before pleurodesis; T2, three hours after pleurodesis; T3, 24 h after pleurodesis; * *p* < 0.05; ** *p* < 0.01.

For the baseline level, patients without successful pleurodesis had a significantly higher pleural fluid TNF-α and IL-10 level before pleurodesis than those who succeeded (Table 2 T1 values and Figure 2). Following the instillation of bleomycin, there was a significant increment of IL-10 in the first three hours in patients who underwent successful pleurodesis. By contrast, there was a significant increment of TNF-α, and IL-10 between 3 and 24 h in patients without successful pleurodesis (Table 3, and Figure 3).

There was no significant difference in the baseline levels or changes following the pleurodesis of IL-1β, IL-6, TGF-β, and VEGF between the patients who underwent successful pleurodesis and those that did not (Table 2 and Table 3; Figure 2 and Figure 3).

### 3.4. Comparison of Pleural Fluid Inflammatory Cytokines and VEGF at Different Time Points within Each Groups

Compared with the relatively lower baseline level, there was a significant increase in IL-10 at 24 h in patients who underwent successful pleurodesis (Figure 2). An initial suppression of TGF-β, followed by a subsequent elevation was noted in both groups, with significant differences in patients who underwent successful pleurodesis.

### 3.5. Correlations between Different Inflammatory Cytokines

Pleural fluid IL-1β and TNF-α was positively correlated before (*r* = 0.726, *p* < 0.001) and at 24 h after pleurodesis (*r* = 0.771, *p* < 0.001). Pleural fluid IL-6 and TGF-β was negatively correlated before (*r* = −0.550, *p* = 0.001) and at 24 h after pleurodesis (*r* = −0.608, *p* < 0.001).

## 4. Discussion

To the best of our knowledge, this is the first study to measure the inflammatory cytokines and VEGF changes following pleurodesis and correlate these factors with the outcome and survival. The possible confounders were taken into consideration (Table 1). The pleural fluid TNF-α levels were significantly higher in the malignant pleural effusion, which may be attributed to the increased local production in the pleural cavity by macrophage, T-lymphocyte, or mesothelial cells upon exposure to inflammatory process [20]. Patients without successful pleurodesis had a higher pleural fluid TNF-α level before pleurodesis. Following pleurodesis, they had an increment of TNF-α in the late stage (between three and 24 h). The tumor necrosis factor alpha is usually regarded as pro-inflammatory and tumorigenic. Deregulation of the TNF-α signaling pathway is associated with many inflammatory disorders, including rheumatoid arthritis, and inflammatory bowel disease, as the monoclonal antibody to TNF-α has been a standard treatment for these diseases. TNF-α have divergent effects on the regulatory T cells, and this contributes to their development and accumulation, although it can downregulate their suppressive capacity in some instances [21,22].

Patients with successful pleurodesis had a significant increment of IL-10 in the first three hours following the instillation of bleomycin. The early surge of IL-10 following pleurodesis accompanied by a longer survival suggests an anti-tumor effect of IL-10. On the contrary, the higher pleural fluid IL-10 levels before pleurodesis and a late increment of IL-10 between 3 and 24 h following pleurodesis in patients without successful pleurodesis suggested the crucial role of IL-10 as a feedback regulator of the increased pro-inflammatory cytokine, TNF-α [12,23,24]. In fact, we also observed feedback regulation in patients who underwent successful pleurodesis when performing intragroup comparison (Figure 2). IL-10 is usually regarded as an anti-inflammatory cytokine and has been reported to exert anti-tumor effects through the increasing tumor antigen-specific CD8+ T cell infiltration and the INF-γ-mediated induction of antigen presentation [12,25,26,27]. Pegylated IL-10 had been developed and demonstrated as an effective anti-tumor immune response with long-lasting immunologic memory [28]. Clinical efficacy has been seen as monotherapy, or in a combination with anti-PD-1 antibodies [29]. However, IL-10 has paradoxical effects on different types of immune response and is considered a potential switcher of immunity [26,27].

The indiscrimination in the baseline levels or changes following pleurodesis of IL-1-β, IL-6, and TGF-β between patients with or without successful pleurodesis was consistent with their reported dual tumor promoting or inhibitory function. Interleukin-6 is regarded as a pro-inflammatory cytokine, although certain anti-inflammatory activities were also attributed to IL-6 [30,31]. The IL-6/JAK (Janus tyrosine kinase)/STAT (signal transducers and activators of transcription) signaling pathway is aberrantly hyperactivated in many types of cancer. Interleukine-6 also exerts immunosuppression in the tumor environment by stimulating the infiltration of myeloid-derived suppressor cells, tumor-associated neutrophils, and cancer stem-like cells. Interleukin-1β activates innate immune cells including antigen presenting cells, and drives polarization of CD4+ T cells towards T helper type (Th) 1 and Th17 cells, to exert anti-tumor effects [32]. Activation of the NLRP3 inflammasome in dendritic cells induces IL-1β-dependent adaptive immunity against tumors [33]. Contrarily, IL-1β within the tumor microenvironment has been reported to promote carcinogenesis, tumor growth, and metastasis through driving chronic non-resolved inflammation, endothelial cell activation, tumor angiogenesis, and the induction of immune-suppressive cells. During intragroup comparison, both groups had an initial suppression of TGF-β, followed by a subsequent elevation, especially in patients who underwent successful pleurodesis (Figure 2). In the early stages of cancer, TGF-β functions as a tumor suppressor, while in the later stages, the TGF-β exerts tumor-promoting effects [34,35,36]. Its effects also depend on the cellular context. TGF-β from the inflammatory tumor microenvironment may cause cancer cell apoptosis and tumor suppression. In contrast, it may also induce an epithelial–mesenchymal transition that promotes cancer cell invasion and metastasis, cancer stem cell heterogeneity and drug resistance [34,35]. TGF-β had been adopted as a sclerosing agent for pleurodesis [37,38], and the anti-TGF-β antibody could inhibit the pleural fibrosis in the rabbit empyema model [39]. Contrarily, the TGF-β inhibitor was proposed as a new line of defense against cancers. Pleural fluid IL-1β and TNF-α were positively correlated, and pleural fluid IL-6 and TGF-β were negatively correlated, both before and 24 h after pleurodesis. The pleiotropic nature of IL-1β or TGF-β makes it a challenging target requiring further study [36].

Similar to the study of Hooper et al. [40], and our earlier study [7], no association existed between the baseline pleural fluid VEGF levels and pleurodesis failure. There was also no association between the changes in pleural fluid VEGF following pleurodesis with the outcome.

The mesothelium itself may regulate the first steps of the pleural fibrosis following the instillation of a sclerosing agent, through the inflammatory response, which is decisive for the pleurodesis outcome and survival [41,42]. Further studies to observe the release of inflammatory cytokines from the mesothelial cells or other cells within the pleural space, such as macrophages after the addition of the sclerosing agent, and the effects of the conditional media, cytokines and their antagonists on cancer cell viability, apoptosis, pyroptosis, proliferation, migration, and invasion are warranted to clarify the mechanism [43,44,45].

The choice of MPE managements, i.e., chemical pleurodesis, indwelling pleural catheter drainage or repeat thoracenteses, depends on the expected survival, lung expandability, and cost-effectiveness, which remains a challenge for clinicians [1,2]. The LENT scoring system appeared to be a valuable prognostic score in patients with MPE [16], as it predicted the shorter survival of patients who failed pleurodesis and may aid clinical decision making in the diverse patient population. The ability to produce specific cytokines in the pleural space after the instillation of the sclerosing agent may be decisive for the pleurodesis outcome and survival. The time points of pleural fluid collection in concordance with the daily practice of pleurodesis in the study allows us to measure the cytokines changes to allocate the patients to adequate treatment strategies in the future. The pleural fluid cytokine concentration 3 h after pleurodesis might be diluted by the amount of saline instilled with bleomycin. This is a drawback and may need be corrected by the protein concentrations in the pleural fluid.

Manipulation of the co-stimulatory or co-inhibitory checkpoint proteins, such as PD-1 and PD-L1, allows for the reversal of tumor-induced T-cell anergy. Cytokines or their specific inhibitors involved in the signaling between the tumor cells and the microenvironment have not, as yet, been systemically studied. In addition to the immune checkpoint inhibitors, the recombinant cytokines can potentially increase the number of patients who will benefit from the immunotherapy [46]. Strategies to target the tumor immunosuppressive network, rather than targeting a single molecule, should be established in the future. In addition, intrapleural cytokine therapy may have the benefit of focused treatment without a systemic effect for patients with MPE [36].

In this study, more patients with symptomatic MPE underwent chemical pleurodesis (33/84, 39.3%), as compared with our earlier series (33.2%) and the historical control (24%) [8,47]. However, there was still an attrition in the subsequent analysis, such as dry drainage that led to missing data at some time points. The tumor heterogeneity of the study group is another concern. To confirm the study findings, such limitations need to be addressed and could be overcome in the future with more and adequate patients recruited.

## 5. Conclusions

The ability to produce specific cytokines in the pleural space following pleurodesis may be decisive for the patient’s outcome and survival. Serial measurement of cytokines can help allocate the patients to adequate treatment strategies. Further study of the underlying mechanism may shed light on cytokine therapies as novel approaches.

## Figures and Tables

**Table 1:** Characteristics of the patients underwent pleurodesis stratified by the results.

Variable	Patients Succeeded Pleurodesis (*n* = 19)	Patients Failed Pleurodesis (*n* = 9)	Patients Survived Less Than 30 Days (*n* = 5)	*p* Value
Mean age, years	55.9 (38.6–74.5)	60.4 (51.2–70.8)	60.6 (48.6–70.1)	0.392
Gender				0.142
Female	16	4	4	
Male	3	5	1	
Smoking history				0.165
Yes	4	5	1	
No	15	4	4	
Pleural fluid LDH level (IU/L)				<0.001
<1500	19	7	4	
>1500	0	2	1	
ECOG PS				0.014
0	1	1	0	
1	14	3	0	
2	4	5	5	
Serum NLR				0.042
<9	15	5	1	
>9	4	4	4	
Tumor type				0.859
Lung adenocarcinoma	9	3	0	
sensitive EGFR (+)	6	0	0	
sensitive EGFR (−)	3	3	0	
breast cancer	10	3	2	
ER or PR or HER2/neu (+)	9	3	1	
TNBC	1	0	1	
ovarian cancer	0	1	1	
small cell lung cancer	0	1	1	
bladder urothelial carcinoma	0	1	0	
gastric cancer	0	0	1	
Total LENT score				0.020
low risk (0–1)	0 (0%)	1 (11.1%)	0 (0%)	
moderate risk (2–4)	19 (100%)	5 (55.6%)	3 (60%)	
high risk (5–7)	0 (0%)	3 (33.3%)	2 (40%)	
mean	2.84	3.67	4.40	

ECOG PS, Eastern Cooperative Oncology Group performance status; EGFR, epidermal growth factor receptor; ER, estrogen receptor; HER2, human EGFR 2; LDH, lactate dehydrogenase; NLR, neutrophil-to-lymphocyte ratio; PR, progesterone receptor; TNBC, triple-negative breast cancer. The LENT score is a validated risk stratification system to predict survival in malignant pleural effusion, calculated on the basis of pleural fluid LDH, ECOG PS, serum NLR and tumor type.

**Table 2:** Measured pleural fluid cytokine and VEGF levels before pleurodesis (T1), 3 hours after pleurodesis (T2), and 24 hours later before the removal of the intrapleural catheter (T3) were compared between patients who underwent successful pleurodesis (*n* = 19) and those that did not (*n* = 12).

	IL-1β (pg/mL)	IL-6 (pg/mL)	IL-10 (pg/mL)	TGF-β (pg/mL)	TNF-α (pg/mL)	VEGF (pg/mL)
T1	*p* = 0.745	*p* = 0.465	*p* = 0.047	*p* = 0.105	*p* = 0.006	*p* = 0.626
Succeeded	0.34 (0.01~58.26)	3351 (14.92~29,484)	10.94 (0.48~63.85)	553.9 (39.00~21,979)	3.19 (0.23~45.85)	179 (2.2~6707)
Not succeeded	1.405 (0.01~3769)	272.8 (30.9~25,967)	18.06 (8.31~116.4)	10,016 (42.16~38,261)	9.58 (3.19~355.7)	486.7 (2.2~1702)
T2	*p* = 0.715	*p* = 0.256	*p* = 0.612	*p* = 0.598	*p* = 0.068	*p* = 0.871
Succeeded	1.75 (0.01~94.33)	3414 (42.99~27,600)	11.12 (0.61~104.6)	174.4 (42.16~6536)	7.83 (0.59~531.2)	231 (9.9~6255)
Not succeeded	2.925 (0.01~114.6)	250.5 (29.05~27,626)	10.86 (3.62~66.38)	174.4 (16.9~32,192)	11.66 (2.44~214.7)	234.3 (2.2~5724)
T3	*p* = 0.372	*p* = 0.516	*p* = 0.209	*p* = 0.273	*p* < 0.001	*p* = 0.626
Succeeded	2.6 (0.005~33.84)	4041 (72.48~30,480)	17 (1.58~250.5)	969.1 (100.5~16,598)	4.15 (0.23~210.5)	393.5 (2.2~5935)
Not succeeded	1.715 (0.1~3953)	773.8 (171.4~29,300)	43.87 (9.76~251.1)	1730 (35.84~25,019)	46.53 (14.48~294.3)	678.2 (2.2~5042)

Data expressed as median and range. IL, interleukin; TGF-β, transforming growth factor beta; TNF-α, tumor necrosis factor alpha; VEGF, vascular endothelial growth factor.

**Table 3:** Fold changes of pleural fluid cytokines and VEGF levels between different time points were compared between patients who succeeded pleurodesis (*n* = 19) and those not succeeded (*n* = 12)**.**

	IL-1β	IL-6	IL-10	TGF-β	TNF-α	VEGF
T2/T1	*p* = 0.068	*p* = 0.516	*p* = 0.008	*p* = 0.105	*p* = 0.273	*p* = 0.776
Succeeded	3.481 (0.073~145.1)	0.95 (0.159~6.986)	1.579 (0.313~8.398)	0.884 (0.003~44.37)	1.272 (0.298~11.59)	0.96 (0.106~44.65)
Not succeeded	1.040 (0.030~11.8)	0.916 (0.323~5.291)	0.511 (0.112~3.68)	0.287 (0.001~64.36)	0.96 (0.007~12.46)	1.002 (0.092~13.85)
T3/T2	*p* = 0.081	*p* = 0.394	*p* = 0.019	*p* = 0.715	*p* = 0.019	*p* = 0.57
Succeeded	0.964 (0.009~38.17)	1.708 (0.203~21.29)	1.718 (0.14~22.52)	1.937 (0.195~289.6)	1 (0.099~10.63)	1.585 (0.087~9.531)
Not succeeded	7.106 (0.463~85)	2.276 (0.978~12.95)	2.831 (0.963~8.818)	3.045 (0.013~345.4)	2.704 (0.403~38.05)	1.244 (0.131~16.62)
T3/T1	*p* = 0.855	*p* = 0.598	*p* = 0.273	*p* = 0.109	*p* = 0.685	*p* = 0.903
Succeeded	4.135 (0.039~88.63)	1.548 (0.182~16.9)	2.576 (0.241~10.96)	0.887 (0.060~128.9)	1.922 (0.072~10.61)	1.452 (0.021~104.4)
Not succeeded	5.75 (0.812~85)	1.941 (0.816~21.71)	1.361 (0.883~7.808)	0.768 (0.087~1.614)	2.072 (0.261~9.734)	1.153 (0.282~19.19)

Data expressed as median and range.
